# Orthotopic heart transplantation associated with hemiarch reconstruction for advanced heart failure and chronic aortic dissection: a case report

**DOI:** 10.1093/jscr/rjag775

**Published:** 2026-10-27

**Authors:** Oscar Edinson Sánchez, Valentina Pérez-Ariza, Roberto H Cruz Buitrago, Juan F Parra, William Rios, Federico Nuñez

**Affiliations:** Cardiovascular Surgery Section, Fundación Clínica Shaio, 111131, Bogotá, Colombia; School of Medicine, Universidad de los Andes, 111711, Bogotá, Colombia; School of Medicine, Department of Surgery, 110231, Pontificia Universidad Javeriana, Bogotá, Colombia; Cardiovascular Surgery Section, Fundación Clínica Shaio, 111131, Bogotá, Colombia; School of Medicine, Department of Surgery, 110231, Pontificia Universidad Javeriana, Bogotá, Colombia; Cardiovascular Surgery Section, Fundación Clínica Shaio, 111131, Bogotá, Colombia; Cardiovascular Surgery Section, Fundación Clínica Shaio, 111131, Bogotá, Colombia; Cardiovascular Surgery Section, Fundación Clínica Shaio, 111131, Bogotá, Colombia

**Keywords:** heart transplant, aortic arch reconstruction, aortic dissection

## Abstract

Concomitant orthotopic heart transplantation and aortic arch replacement are extremely rare, particularly in chronic Stanford type A dissection. A 43-year-old man with end-stage heart failure and chronic aortic dissection underwent combined orthotopic heart transplantation and hemiarch replacement. Because of concomitant liver donation, the donor inferior vena cava required pericardial patch reconstruction. Despite early right ventricular dysfunction requiring veno-arterial ECMO, the patient recovered completely and was discharged with preserved graft function. This case demonstrates the feasibility of a single-stage combined approach with excellent postoperative outcomes in carefully selected patients.

## Introduction

Concomitant heart transplantation and replacement of the ascending aorta and aortic arch is extremely infrequent. In the few cases reported, most patients were diagnosed with Marfan syndrome and had an aortic aneurysm [1, 2]. The combination of these procedures, because of a Stanford A dissection, has only been reported on two occasions, with only one involving the aortic arch [3, 4]. End-stage heart failure in the setting of complex aortic pathology presents a unique surgical challenge. Therefore, we present a case of a 43-year-old who was taken to a combined orthotopic heart transplantation and hemiarch replacement because of a chronic Stanford A aortic dissection. This case highlights the feasibility of a single-stage approach under meticulous surgical planning and postoperative support.

## Case presentation

A 43-year-old man presented with progressive dyspnea, he had a known diagnosis of heart failure with reduced left ventricular ejection fraction (LVEF), chronic aortic dissection involving the ascending aorta (Stanford type A) as shown in Figs 1 and 2, severe aortic regurgitation, and moderate mitral regurgitation. On examination, he was clinically stable but exhibited signs of systemic congestion. Echocardiography revealed a markedly dilated left ventricle with eccentric hypertrophy, severely depressed systolic function (LVEF 16%), moderate-to-severe mitral and severe tricuspid regurgitation, and biatrial dilation. The patient was presented in the transplant board and was therefore listed for urgent cardiac transplantation (0A–VIII). The initial surgical plan considered a combined heart transplantation with ascending aorta reconstruction. It was initially proposed to evaluate the possibility of retrieving the donor’s aortic arch to allow for an extended replacement of the recipient’s dissected arch. However, after further discussion, performing the aortic dissection repair first (under deep hypothermic circulatory arrest) was considered more feasible and with lower surgical risk. The operative strategy included starting the procedure in the recipient, performing the dissection repair, and initiating donor organ extraction once rewarming began, in order to minimize total ischemic time.

**Figure 1:**
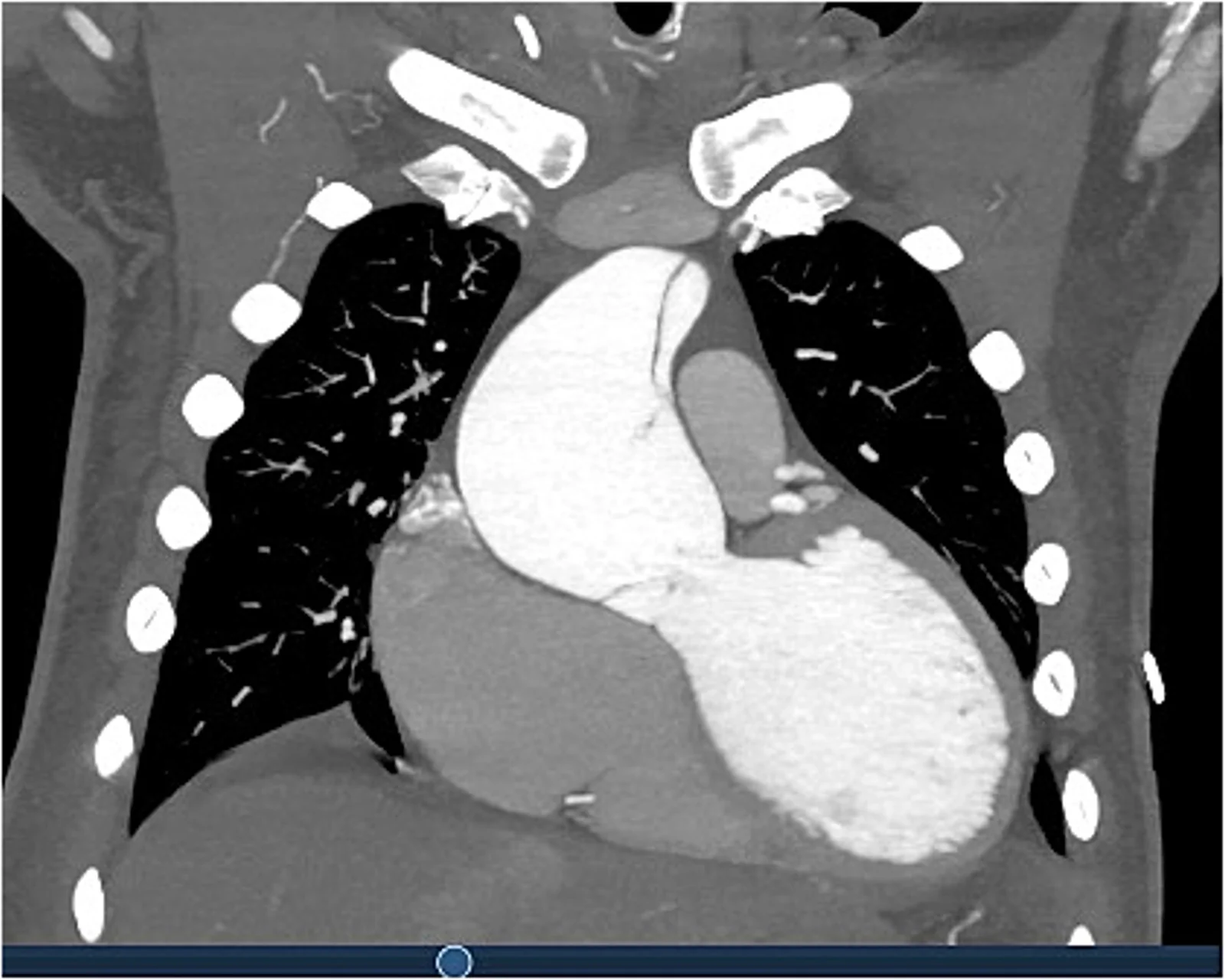
Coronal contrast-enhanced computed tomography demonstrating a Stanford type A aortic dissection. The image shows a clear intimal flap originating in the ascending aorta and extending into the proximal aortic arch, separating the true, and false lumens.

**Figure 2:**
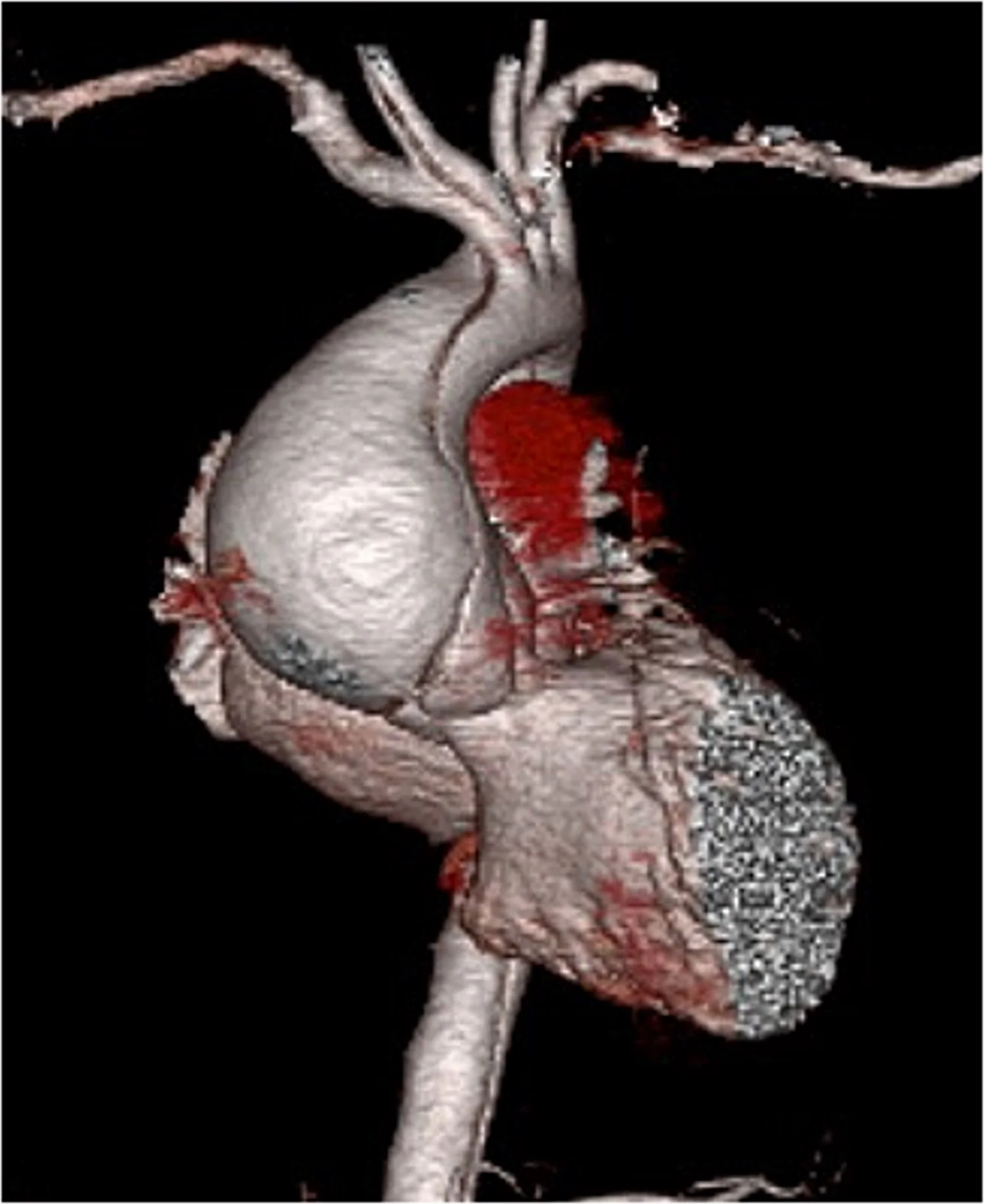
Three-dimensional volume-rendered reconstruction of the thoracic aorta demonstrating a prominent intimal disruption with formation of a false lumen. The true lumen appears compressed posteriorly. The dissection flap propagates toward the aortic arch, with partial involvement of the arch curvature.

Although the initial plan was to follow the proposed preoperative plan, the donor developed hemodynamic instability, which precluded waiting until completion of the aortic reconstruction to initiate extraction. The procedure was therefore performed through a median sternotomy under general anesthesia and cardiopulmonary bypass. After systemic heparinization, femoral arterial and bicaval venous cannulation were done. Following initiation of bypass, the ascending aorta was cross-clamped. The native heart was excised, and under deep hypothermic circulatory arrest at 20°C, the ascending aorta and proximal arch were resected, and a 28-mm Dacron graft was interposed distal to the innominate artery to reconstruct the hemiarch. A secondary 8-mm side graft was anastomosed end-to-side to the main conduit to re-establish arterial perfusion.

During implantation, the inferior vena cava of the donor graft was found to be short, as the donor was also a simultaneous liver donor, limiting the available length of the caval cuff. To achieve a tension-free anastomosis, the recipient’s inferior vena cava was reconstructed with a heterologous pericardial patch. The donor aorta was sutured to the Dacron graft (Fig. 3). The total cardiopulmonary bypass time was 315 min, cardiac arrest time 22 min, and ischemic time 192 min.

**Figure 3:**
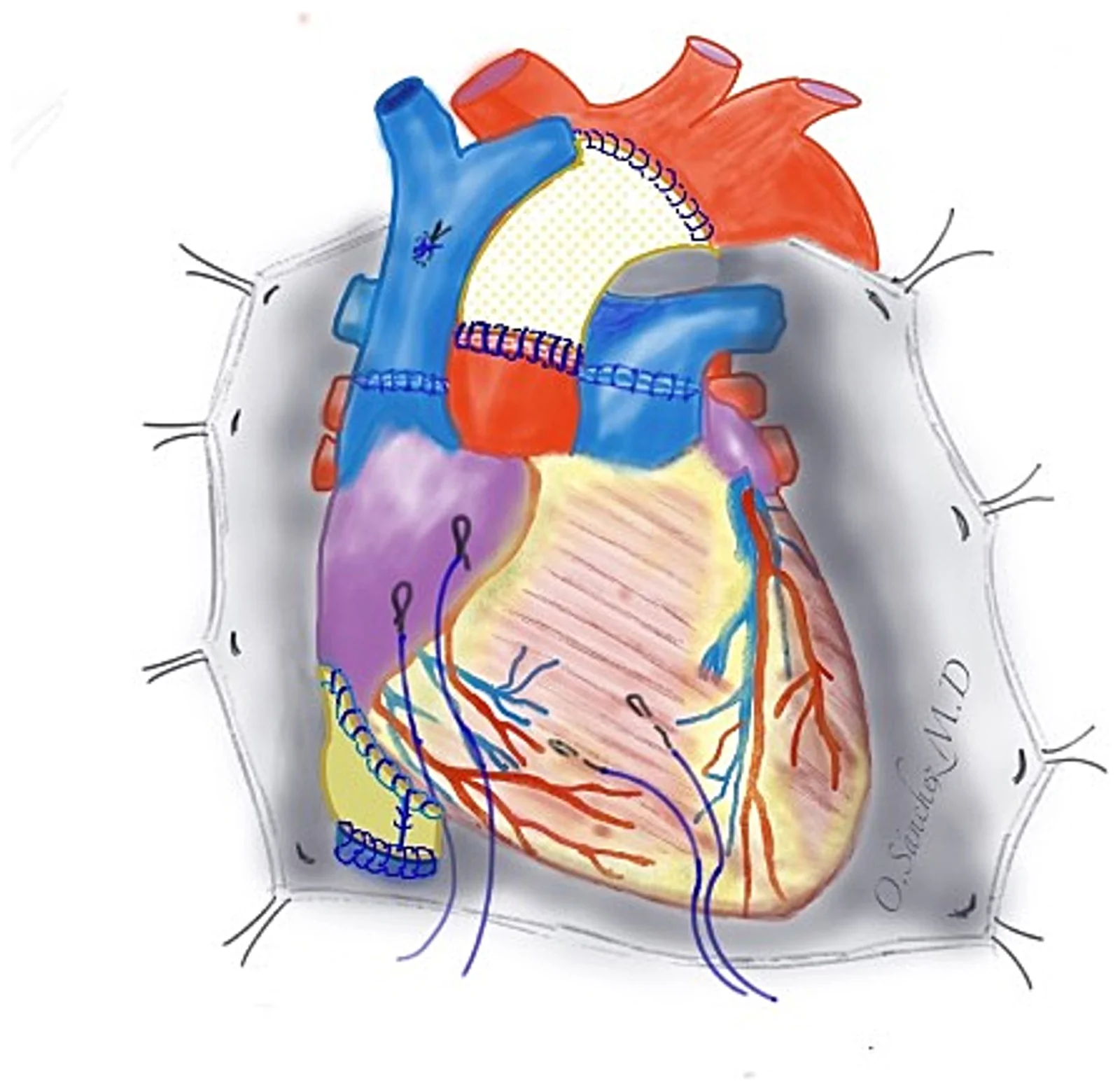
Schematic representation of the operative technique performed during combined orthotopic heart transplantation aortic arch and inferior vena cava reconstruction.

Because of persistent right ventricular failure, central veno-arterial ECMO was placed. Progressive myocardial recovery over 48 h allowed successful weaning and decannulation on postoperative day 2. He subsequently developed a pericardial effusion requiring drainage on postoperative day 8, yielding 1300 ml of old hemopericardium without active bleeding. Thereafter, recovery was uneventful. The patient started on a triple immunosuppressive regimen on postoperative day 5. He was discharged in good clinical condition with preserved graft function (LVEF 68%). Endomyocardial biopsy was performed at one week and two weeks postoperatively, and both showed no evidence of acute cellular or humoral rejection.

## Discussion

Combined orthotopic heart transplantation with hemiarch and ascending aorta replacement in the context of a chronic Stanford type A dissection is exceedingly rare, with only two cases described in the literature, and only one involving the aortic arch [4, 5] This combination substantially increases surgical complexity and perioperative risk [2, 6].

Earlier reports demonstrated the feasibility of performing orthotopic heart transplantation with ascending aortic or hemiarch replacement under hypothermic circulatory arrest, achieving favorable long-term outcomes [1, 7]. It is well known that for these cases meticulous preoperative planning and precise cerebral protection strategies allow safe arch reconstruction without excessive ischemic times [4, 6].

Our case shows an innovative technique by integrating hemiarch replacement and orthotopic heart transplantation in a patient with chronic aortic dissection and severe left ventricular failure. This described technique has not been reported previously in the literature. This surgery, performed under deep hypothermic circulatory arrest, achieved satisfactory ischemic (192 min) and bypass (315 min) times, comparable to those reported in prior cases (205–351 min) [8].

Although ECMO support was initially intended to be avoided, it became necessary due to early graft dysfunction, predominantly affecting the right ventricle. This highlights the value of temporary mechanical support as a bridge to recovery in technically demanding combined procedures.

## Conclusions

This case demonstrates that concomitant orthotopic heart transplantation and aortic hemiarch replacement, although technically demanding and associated with increased operative risk, can be successfully performed in carefully selected patients. Meticulous preoperative planning, precise surgical sequencing, and the judicious use of temporary mechanical support were key to achieving a favorable outcome.
